# Preventive Conservation of a Short Theatre Skit (Valencian “Sainete”) with Cloud Data Storage and Internet of Things

**DOI:** 10.3390/s23249790

**Published:** 2023-12-13

**Authors:** Irina Verdesoto, Ángel Fernández-Navajas, Pedro Javier Beltrán-Roca, Fernando-Juan García-Diego

**Affiliations:** 1 Facultad de Artes, Universidad Central del Ecuador, Quito 170129, Ecuador; imverdesoto@uce.edu.ec; 2 Polytechnical Department, School of Architecture, Universidad Europea de Canarias, 38300 Tenerife, Spain; angel.fernandez@universidadeuropea.es; 3 Beltran & Ingenieros Asociados S.L.U, 46008 Valencia, Spain; pjbeltran@beltraningenieros.es; 4 Departamento de Física Aplicad Escuela Tecnico Superior de Ingenieros Industriales, Universitat Politècnica de València, 46022 Valencia, Spain

**Keywords:** preventive conservation, time capsule, Valencian “sainetes”, microcontroller, relative humidity, temperature, online platform

## Abstract

Preventing the progressive deterioration of works of art over time is a topic of great interest to collectors and museums. With this aim, time capsules where environmental conditions remain unchanged are well known for preserving art. In this paper, a prototype of an IoT time capsule is presented with a focus on low cost in order to make it accessible to private collectors or small museums with tight budgets. Valencian ‘sainetes’ (small plays), which are considered materials of artistic interest, have been placed in a “time capsule”, which is a manually made container with insulating materials for keeping small pieces for a long time. Environmental control has been performed with a low-cost microcontroller, sensors and actuators connected to a free online IoT platform. This platform recorded data and made decisions based on these data, sending cooling or heating orders to an environmental control system. The results obtained are very satisfactory and open interesting perspectives for future research. However, they also highlight some relevant technical and economic limitations that will have to be considered in the future.

## 1. Introduction

Preventive conservation is the part of the science of the restoration of cultural heritage that studies how to preserve works of art to extend their lives and make their conservation and intervention, when required, more economical.

Currently, large museums are implementing very sophisticated preventive conservation plans based on microclimatic monitoring. However, works of art which are not considered/deposited in modern, purpose-built museums or those that could often be found in their original location, such as ancient buildings, churches, small collections and/or private collections, lack such plans.

The conservation of our heritage attempts to preserve a small collection that may be of local or even family significance, but that also may later be an important piece in explaining the culture or way of life of our ancestors.

The historical way of preserving cultural artefacts has been through the fortuitous creation of sites with favorable climatic conditions, either naturally controlled or constant. This method is called “time capsules”, which can, for instance, be a desert (Tutankhamun’s tomb), a cave (Altamira) or Pompeii. Since these sites were discovered, their development has led to their rapid degradation [1,2,3], and even their study is a scientific challenge [4], with chemical reactions never before described by science being discovered [1,2].

The term “time capsule” refers to boxes made of insulating materials where memories such as photographs, toys or written notebooks are stored, and after some time, they are rediscovered and opened.

With these premises, we could consider those pieces as a fortuitous, unique and unrepeatable experiment.

For centuries, mankind has tried to create “time capsules” to preserve the “soul” or eternal life [5]. In the case of Cuban cigars, they are packed in boxes with a microclimate constant of 16 °C and 18 °C, because if the temperature increases, it favors the formation of microorganisms, bacteria and parasites [6]. And more recently, such “capsules” have been found in Madrid, as reported by the General Directorate of Historical Heritage. Of the time capsules that have been found, the last one was buried under the statue of Cervantes in the Plaza de las Cortes, which could originate from 1835 and has been moved to the Regional Archaeological Museum, and another was buried under the statue of Isabel II located in Opera, inside which there are “several coins, diaries of the time and a copy of the minutes of the inauguration ceremony of the same stature, among other objects” [7].

Today, modern methods are used for preventive conservation, and states have formulated standards for this purpose. The first Italian standards [8,9] set an appropriate constant temperature and humidity. Subsequently, the European Union updated these standards [10] on the assumption that, if a work of art has reached the present thanks to variable climatic conditions, it should be able to be preserved over time if these conditions do not change. These conditions can be a high humidity and an average temperature [3] or a high temperature and a low humidity [7]. This also makes the preservation of works of art more energetically sustainable.

The European Union is aware that the diffusion of its culture and heritage represents an important economic income. In an attempt to monopolize historical cultural tourism and the spreading of history with a certain hegemonic vision, investment in projects to conserve small and medium-sized collections has been performed [11,12,13] through H2020. These projects are based on the premise of conserving in vines [12] to facilitate the maintenance of the temperature and humidity of the piece, using the Internet of things (IoT) to monitor the microclimatology, warning restorers of possible problems in the preventive conservation of the piece [13], or the use of specific containers for the protection of very particular and new pieces in heritage, such as cellulose support for films, photographs, etc. [11].

IoT (the Internet of Things) is a term used when we talk about objects that send information over the Internet to a database located on an external server. This information comes from sensors that take readings of some physical parameter related to an object, such as temperature, CO_2_ level, etc.

The use of data loggers for cultural heritage monitoring is widely discussed in the literature [14,15,16,17,18,19,20,21]. Other uses for these systems are also found in other disciplines, with ingenious ideas such as air quality monitoring in large cities [22].

Brand owner data loggers such as Hobo data loggers [15,16,23], DS1922L [18] and DS1923 [19] have been used [17,18,21]. Hardware and software from these data loggers are undisclosed and cannot be modified, and these data cannot be delivered easily and automatically to the cloud for their manipulation and study.

Subsequently, wired data acquisition systems [24] were developed to be suitable for the conditions of museums and the monitoring of large works of art, where a lot of data from remote points were stored in a single database [25]. The next advance in heritage data acquisition was the hybrid (wired/wireless) data logger [26], giving more versatility to the data acquisition system. The problem with all those systems is that the data must be physically collected.

The next step would be to send out the data to the cloud [13,27] in order not to travel. A final step [13,27] would be to be able to make decisions for curators in the cloud.

These last two systems have several disadvantages. As it is a system with a low production of nodes, its price will be high. By using third-party electronic components, you are exposed to the end of production. The battery must have a high density, and its catastrophic failure could cause irreversible deterioration of the work to be protected. Being a closed system, the adaptation and/or change of some parameters requires a specialist.

In this work, for instance, we develop a time capsule with dedicated software and cloud connectivity, specifically designed for preserving Valencian ‘sainetes’ (Valencian sketches).

There are records of Valencian “sainetes” from the 18th century to the present day. The Interuniversity Institute of Valencian Philology, which brings together the Universidad de Alicante, the Universitat Jaume I and the Universitat de Valencia in research work, has produced the Computerized Bibliographic Inventory of Valencian Popular Theatre of the Beginning of the 20th Century (1917–1950) [28], which is a sample of material wealth and local tradition, containing information on 2706 works by 684 authors [28].

“Sainetes” are short plays that include “indications (...) for performance and provide significant data for its understanding (real or fictitious place of development), list of characters, scenery, (music) and notes” [29]. According to this author, some of them ceased to be monologues to become a more complex comic act with several characters that were performed on a stage, in the town squares or in the neighborhoods of Valencia. They have been classified according to their plots as “falleros”, “fogueros” or “fiesteros”. As objects of popular theatre, they have been difficult to catalogue and preserve, as they are handwritten, typed or poor-quality publications. Most of them were written in Valencian before the language normalization in 1992. According to [29], the posters of the performances of these “sainetes” have hardly been preserved. In them, we could have found the most accurate information about the variety of titles, authors, performers who played them or scenic spaces; and moreover, many of the texts of the plays that were performed have not been preserved either, since they were not well received by the public and were never published.

Currently, there is a commercial technology capable of creating a “time capsule” to preserve works of art belonging to low-cost culture expressions, which has an intelligence tool in the cloud to maintain the temperature, humidity and other chemical parameters, such as temperature (T), relative humidity (RH), CO_2_ and VOCs, in a stable and controlled way at all times, which are measurable and useful for the preventive conservation of small or medium-sized collections of documents of popular culture such as Valencian “sainetes”.

To achieve this goal, a low-cost “time capsule” is built with the intelligence to regulate the hardware shared in situ and in the cloud. This intelligence controls temperature and humidity and monitors other chemical parameters, according to international paper conservation standards. In turn, all data and alarms will be located in the cloud. This capsule could be scalable and useful for the preventive conservation of small or medium-sized collections of documents or any other type of ancient piece of our cultural heritage.

It is essential to emphasize the concept of “low cost”, as there are currently various commercial systems on the market that carry out localized environmental monitoring and even make it possible to change the thermo-hygrometric conditions to keep them within limits that guarantee conservation. However, these systems are complex and expensive, which is a barrier to access for small collectors and even for modest institutions, public or private, that treasure art pieces of great value but possess tight budgets.

## 2. Materials and Methods

The conservation chamber is composed of (1) an insulated container where the items (in this case, the “sainetes”) are deposited for their conservation; (2) local hardware and software capable of controlling (i) temperature and humidity, (ii) a real-time clock (RTC) and (iii) a micro-SD for saving climatic data; and (3) cloud software where a complementary set of data is to be saved and alerts to curators could be implemented.

A lot of studies, summarized in [23,30], have investigated the microclimate data collected in libraries, considering several standards and guidelines for heritage conservation. According to these studies, we chose 22 °C for the fixed temperature and 60% for the relative humidity (RH). To reach the chosen value of RH, we closed the conservation chamber when the outside RH was enough to provide 60% when the chamber reached 22 °C.

### 2.1. Time Capsule Description

**Figure 1:**
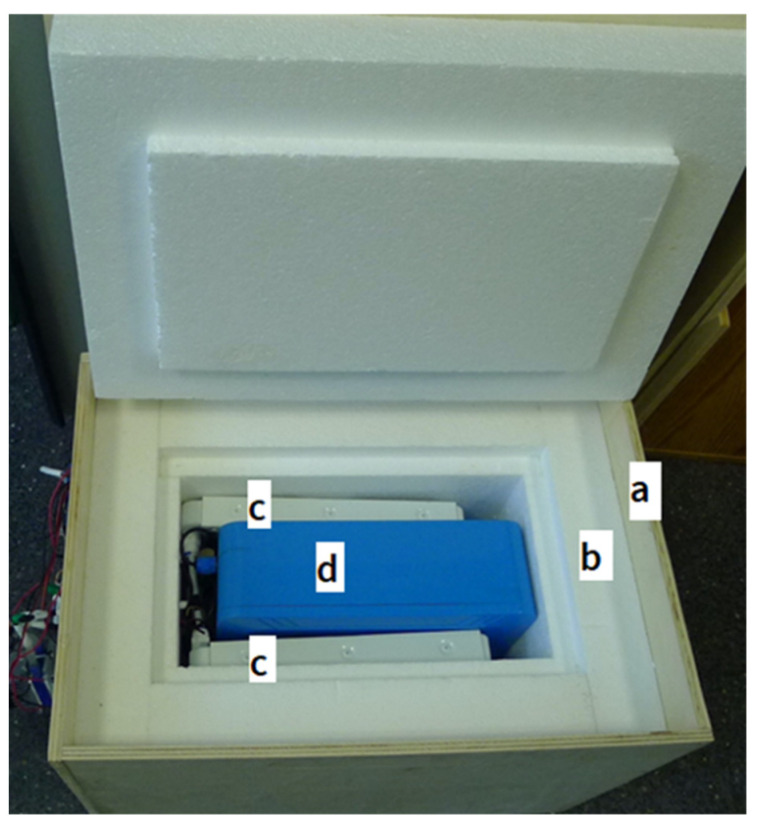
The time capsule is composed (from exterior to interior) of (a) laminated wood; (b) polystyrene; (c) two water radiators; and (d) a hermetic plastic box.

Figure 1 shows the complete time capsule.

The external dimensions are 50 cm × 38 cm × 35 cm.

The container is made of the following materials:

An outer box with a lid made of two layers: an outer layer of wood (10 mm thick) (Figure 1a) and an inner insulating layer of white cork (60 mm thick) (Figure 1b).

A Hermetic IP68 waterproof plastic box for electrical projects, with the following dimensions: 290 mm × 210 mm × 95 mm (Figure 1d).

The hydraulic circuit consists of

A 5-volt water pump with a USB connection.

Two Peltier cells (TEC1-12706) of 12 V and 60 W each with heat sinks.

Two 120 mm × 240 mm × 36 mm aluminum radiators, with 18 tubes (Figure 1c).

A ¼” pipe with elbows and adapters.

**Figure 2:**
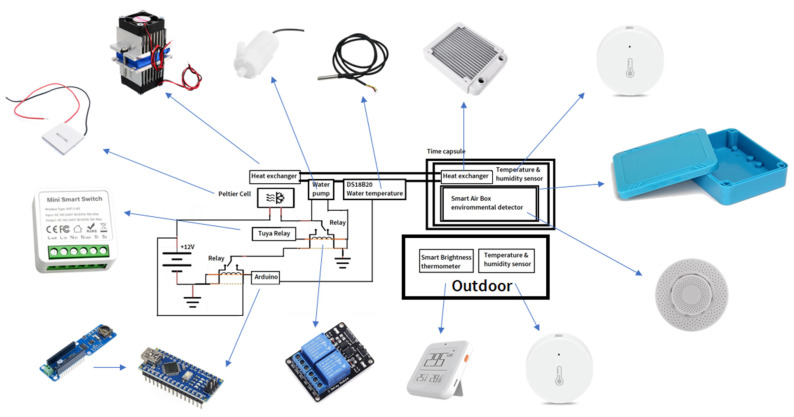
Electric diagram and photographs of all components used to build the “time capsule”.

An electric diagram and photographs of all the components used to build the “time capsule” can be seen in Figure 2. An attempt has been made to ensure that all the local electronic components are as standard as possible. The smart sensors were chosen for their compatibility with the TUYA website. The unitary price of the components and the total cost of the time capsule are shown in Table 1.

All components were purchased directly from Chinese distributors. These distributors do not provide model names in most sales; they only provide the technical data listed in Table 1.

### 2.2. Cloud Storage

In this work, the services of the TUYA platform (https://www.tuya.com/ (accessed on 3 November 2023) were used. This platform, explained in a simplified way, provides a data storage system in the cloud, accessible by any device connected to the Internet. Devices can send data to this service through a protocol provided by TUYA, and the platform is responsible for storing and analyzing them, and depending on the conditions set by the user, called scenarios, it will emit signals that will be received locally by a microprocessor that will perform the action established for those conditions through actuators, for example, opening a relay, turning on a light, or starting the motor of a cooling system.

These platforms are the basis of IoT projects and have advantages and disadvantages that must be studied in detail to choose the one that best suits the needs of the project.

The advantages include their ability to store large amounts of data and provide great power to perform calculations or searches remotely. As the computing power resides in the cloud, there is no need to install advanced computers locally; small, inexpensive microcontrollers connected to the Internet can be used, which only need to send data from the sensors and receive commands to drive the actuators.

Another advantage is that the data can be accessed from any device connected to the Internet (PC, mobile phone, tablet) as long as you have the appropriate permissions.

On the other hand, these services make constant use of remote servers, so they require the payment of fees for the time they are used. Sometimes they also require the sensors to be compatible with their acquisition system, so they offer commercial packages that include the use of their dataloggers and the payment of a monthly or annual fee for the use of their services.

This means that, in many cases, these commercial solutions are very costly, which makes their use unfeasible for all users.

For example, Testo^®^ (www.testo.com, accessed on 22 November 2023) offers a datalogger model (Wi-Fi testo 160 THL) focused on preventive conservation in heritage to record temperature, humidity, light and UV radiation that, with accessories, has a cost of EUR 800 approx., and it is necessary to pay an annual fee of EUR 23 for each datalogger that is installed [31].

There are platforms that sometimes have commercial promotions and offer free plans with limited storage and functionality. In our case, TUYA offered, for a few months in 2021, a free plan that allowed data to be sent and stored for only 10 days, after which the data were deleted. It also allowed data to be sent from any sensor, not just TUYA. This plan fit with the required limitations of our project and was the one chosen to perform this work.

Another important issue when using these platforms is the privacy and security of the data sent. Regarding privacy, it should be noted that the data collected in these projects are not highly sensitive, and if detailed descriptors are not used, any theft would make them useless to outside observers. On the other hand, bearing in mind that no system, whether local or online, will ever be 100% secure, it can be considered that a good Internet server, correctly configured by specialized personnel, will always have an adequate level of security.

**Figure 3:**
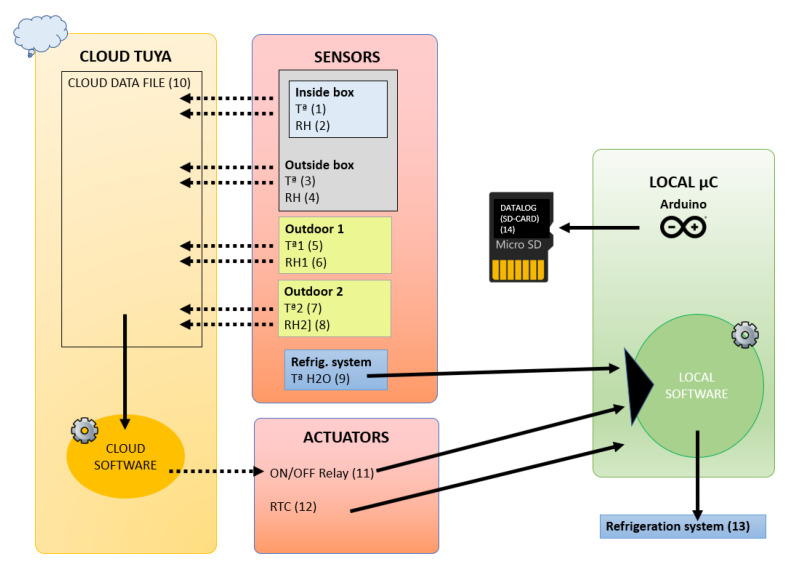
Hardware schema of the “time capsule”. Dashed arrows indicate when information travels through the cloud, wirelessly. Continuous arrows indicate that the information travels locally.

In spite of this and following the principle of prudence, it is always advisable to keep a copy of the data collected in a local system. The system proposed in this work, in addition to sending the data to the platform, saves a local copy of the data on an SD card (Figure 3 (14)). This increases the security against data loss.

### 2.3. Local Hardware

The sensors (1–8) in Figure 3 are commercial sensors that are TUYA compatible, and they have the capability of connecting to the TUYA web and to its app.

No modifications have been made to these sensors. The technical specifications are shown in Table 2:

All these sensors send data to the free TUYA cloud, and scenarios can be defined by the user with the app. Also, the data from all the sensors are automatically stored in the cloud. In the free version, only the data for 10 days are stored. 

The Smart Air Box environmental detector also reads and stores in the cloud the concentration of formaldehyde, volatile organic compounds (VOCs) and carbon dioxide (CO_2_). Furthermore, the Smart Brightness thermometer may also read and store illumination conditions in the cloud. These parameters could be very useful for preventive conservation, but at the moment, they are out of the reach of this investigation.

### 2.4. Cloud Software

**Figure 4:**
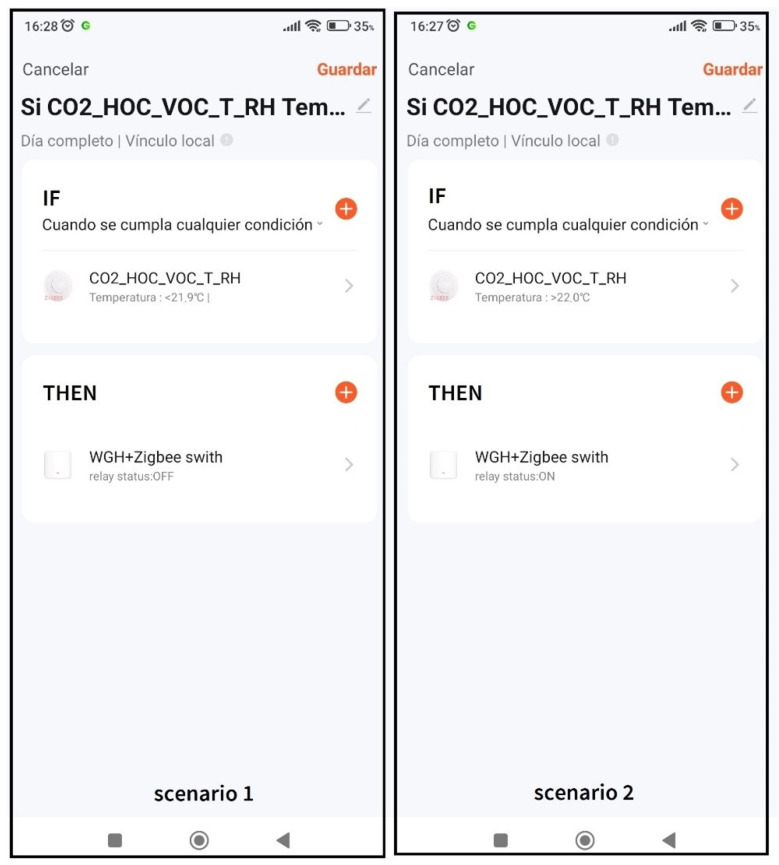
TUYA application from which the scenarios are created.

From the TUYA app, two scenarios for cooling have been created, as seen in Figure 4.

SCENARIO 1:
  IF inside box temperature Figure 3 (1) is lower than 21.9 °C. THEN the ON/OF relay of Figure 3 (11) is in OFF position.

SCENARIO 2:
  IF inside box temperature Figure 3 (1) is greater than 22 °C. THEN the ON/OFF relay of Figure 3 (11) is in ON position.

### 2.5. Local Software (Arduino)

A “microcontroller” is an integrated circuit with input and output pins (digital and/or analog) that are able to communicate with the physical world through sensors and/or activate actuators according to a single program created by the user.

An Arduino microcontroller was used since it is free software and hardware. This microcontroller reads the temperature of the water radiator cooling system (Figure 3 (9)) through a 1-Wire temperature sensor type DS18B20.

Every second, the system reads the temperature sensor (Figure 3 (9)), and if it is higher than 22.1 °C and the ON/OFF relay (Figure 3 (11)), which is controlled by the cloud software, is ON, it turns on the pettier cell to cool the water and the recirculation pump of the hydraulic circuit (Figure 3 (13)). The water cooling system only stops when the water temperature is below 21.9° or the ON/OFF relay (Figure 3 (11)) is OFF.

In addition, the system reads the real-time clock (RTC) (Figure 3 (12)) and stores the time, the relay ON/OFF status (Figure 3 (11)) and the cooling water temperature (Figure 3 (9)).

## 3. Results and Discussion

### 3.1. Overview

The results obtained show the viability of the system and the validation of the idea that it is possible to maintain optimal conditions for the conservation of small pieces of art at affordable costs for any collector or small museum. Furthermore, it is possible to use current technologies, such as the cloud storage of data and the use of intelligence through the internet, while maintaining low costs.

### 3.2. Temperature

**Figure 5:**
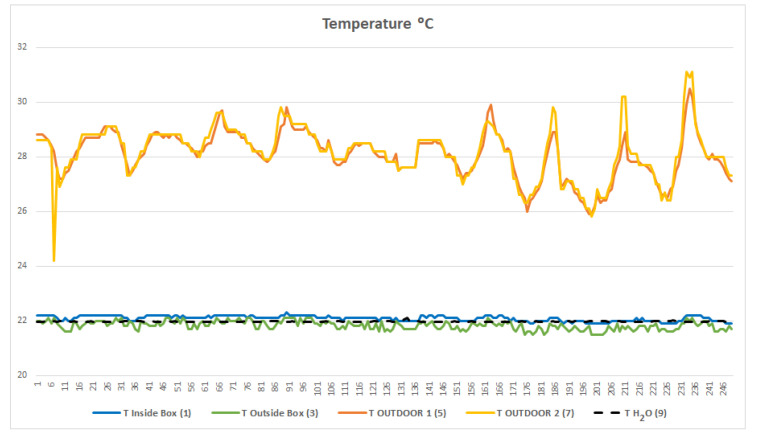
Graph of temperature progression. The *x*-axis indicates the time of the monitoring period. To identify each sensor, the nomenclature used in Figure 3 is indicated in parentheses. Note how the cooling system’s water temperature remains constant (indicated in dashed black line).

Figure 5 shows the uniformity of the water temperature of the cooling/heating system throughout the monitoring period. As explained above, the cloud platform receives the inside container temperature data, and if it falls outside preset limits, it sends a cooling or heating order to the microprocessor in charge of the cooling circuit. Since the experiment was carried out in the middle of summer, the indications were always to cool down the system.

**Figure 6:**
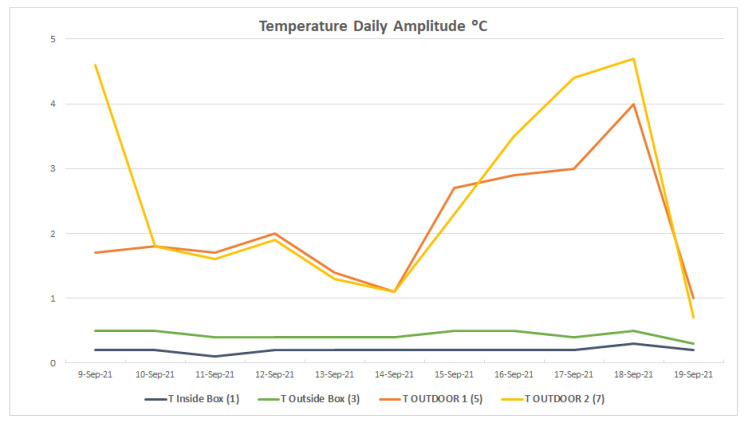
Graph of the temperature’s daily amplitude. Daily amplitude is the difference between the maximum and minimum temperature value over 24 h. It is an indicator of how fast changes in the climate parameter, in this case, temperature, are occurring. A value of 5 indicates a difference of 5 °C between the two extreme values. Values close to zero indicate that the parameter remains constant, which is favorable for preventive conservation.

Thanks to the constant temperature of the water circuit and the environmental insulation, the temperature of the container is also kept constant, with daily variations of less than 0.5 °C, while the outside temperature varies up to almost 5 °C (Figure 6).

### 3.3. Relative Humidity

**Figure 7:**
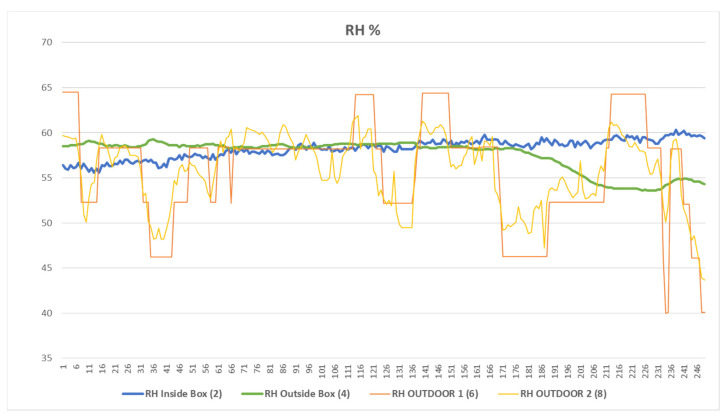
This figure shows the values of the ambient RH versus the container’s RH where the artworks were stored. The variations in the outdoor RH, compared to the variation values of the containers, were remarkable.

**Figure 8:**
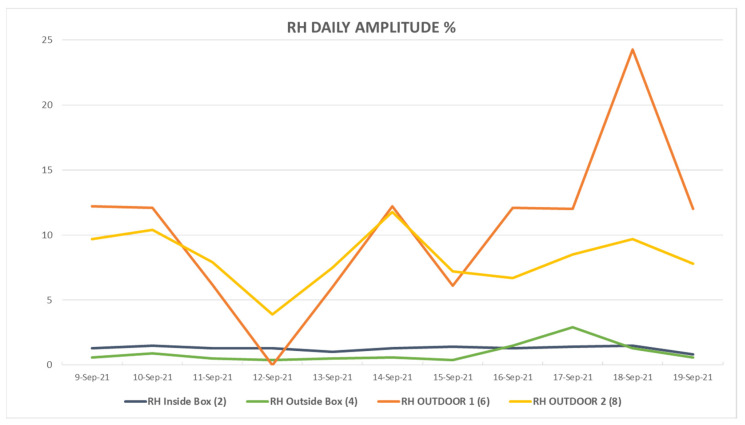
Daily amplitude of RH, which is calculated as the difference between the maximum and minimum values over 24 h. In the same way as for temperature, this figure quantifies the variations, and it can be seen that these variations are very low (close to zero) in the containers where the pieces are kept.

As the temperature remains constant inside the containers, since they are insulated and no exchange of water occurs, the RH also remains constant, as can be seen in Figure 7. Figure 8 quantifies these variations; while the RH outside suffers daily variations of around 15%, the variation in the RH inside the box has a maximum of 1.5%.

### 3.4. Drawbacks of the System

Inexpensiveness, which marks the whole philosophy of this prototype, also imposes some limitations that cannot be ignored and must be considered.

As mentioned above, a cloud platform has been used to receive and store data and to provide intelligence by sending cold/heat signals to the cooling circuit. Logically, this platform has operating costs, and its use requires the payment of a fee. In this case, however, a free plan was used, which limits free data storage to 10 days. After that time, all data are deleted and no longer available, and storage starts again for another 10 days. Unfortunately, the cost of the cheapest non-free plan is inaccessible for our purposes. This forces us to deal with the limitations of the free plan and to save the data every 10 days.

Those were the conditions under which data were recorded during August and September 2022. At present, the conditions of the free plan have changed and only allow data to be stored for 5 days, and the data cannot be downloaded. With these new conditions, the usefulness of the platform is practically null, as it does not allow data to be collected for further study.

This has happened in other similar projects [13]. The bankruptcy of the company Sigfox, a partner of Collection Care, put the entire project in check. This company was in charge of providing the technology to send data to the cloud, where it should be processed.

Another drawback was the use of cheap sensors, which sometimes leads to low sensitivity. However, this has not represented a major problem as seen in the collected data and can be partially overcome by using several sensors.

The conceptual idea of this work is not new. Researchers at the Universitat Politècnica de València (Spain) developed a monitoring system for works of art that ultimately failed to achieve the expected objectives [13,27] due to changes in the external technology used.

The industry also has numerous technical solutions for measuring environmental parameters, although these systems are not specialized in monitoring works of art. Some commercial companies that develop environmental measurement systems include Testo, already discussed above, Enviraiot [32] and Sensonet [33]. All of them offer customized solutions that include dataloggers and sending data to their online platforms, as well as analytics and alarms. Their drawback, as explained above, is that they tend to be very expensive and require high fees to use IoT services. This makes them unfeasible for private collections and small museums.

Therefore, the differential point of this work is to bring this technology to any work of art held by small collectors through the use of low-cost sensors and, subsequently, to develop a free online platform for preventive conservation.

## 4. Conclusions

The results obtained allow us to affirm that the thermo-hygrometric conditions of the artwork containers, especially the conditions of the inside box, which benefits from physical insulation and the regulation of the heating/cooling system, are excellent for the preservation of the artworks.

These results highlight the possibility of implementing low-cost systems that are accessible to small collections and guarantee the appropriate environmental conditions for their conservation.

The time capsule prototype developed in this work demonstrates that the idea of the low-cost preventive conservation of any work of art, no matter how small, and no matter how small the owner’s budget, is feasible.

However, this is only the first step: a hardware tool that needs to send the collected data to an online platform to store and process all the information. This work has highlighted the strong limitations of the free plans of the platforms, which are nothing more than a commercial lure to test the services for a very short period of time, which is totally insufficient to collect a minimal amount of scientifically valid data. And unfortunately, on many occasions, the non-free plans have unaffordable economic costs. This work could be carried out because the objective was to demonstrate the feasibility of the idea without pretending to carry out a complete monitoring over several months or years.

With all these limitations, there is a need to develop, based on this low-cost hardware, a free IoT platform hosted by non-profit entities (universities, for example), open to any artwork owner, and breaking the limitations of commercial platforms as well as their cost. An example of this would be a platform developed by multidisciplinary teams made up of scientists, computer programmers and art specialists.

## Figures and Tables

**Table 1:** Cost of the components of the “time capsule”.

Commercial Name	Uds	Price
Smart Air Box environmental detector	1	EUR 27.02
Temperature and humidity sensor	1	EUR 0.99
Smart Brightness thermometer	1	EUR 24.03
Temperature and humidity sensor	1	EUR 0.99
DS18B20	1	EUR 0.62
Arduino nano	1	EUR 6.57
Arduino shield datalogger	1	EUR 2.77
Water Pump5V USB	1	EUR 1.98
Peltier modules TEC1-12706, 12 V, 60 W	1	EUR 1.92
Two 120 mm × 240 mm × 36 mm aluminum radiators, with 18 tubes	2	EUR 11.12
Heat exchange	1	EUR 18.09
IP68 waterproof plastic box for electrical projects	1	EUR 21.60
Other hardware (wood, pipes, etc.)	1	EUR 30
Tuya relay	1	EUR 1.25
Two-channel relay	1	EUR 1.86
TOTAL		EUR 155.36

**Table 2:** Technical specifications of sensors.

Commercial Name	Location	Number	Measured Parameter	Range	Accuracy
Smart Air Box environmental detector	Inside Box	1	T (°C)	0–60	-
		2	RH (%)	0–95	-
Temperature and humidity sensor	Outside Box	3	T (°C)	−20–50	±0.3
		4	RH (%)	0–100 (no condensation)	±3
Smart Brightness thermometer	Outdoor 1	5	T (°C)	0–50	±0.3
		6	RH (%)	0–99	±3
Temperature and humidity sensor	Outdoor 2	7	T (°C)	−20–50	±0.3
		8	RH (%)	0–100 (no condensation)	±3
DS18B20	Refrig.system	9	T (°C)	−55–125	±0.5

## Data Availability

Data supporting reported results can be found by contacting the authors.

## References

[1] Sandström M., Jalilehvand F., Persson I., Gelius U., Frank P., Hall-Roth I. (2002). Deterioration of the seventeenth-century warship Vasa by internal formation of sulphuric acid. Nature.

[2] Pérez-Diez S., Martí A.P., Giakoumaki A., Prieto-Taboada N., de Vallejuelo S.F.-O., Martellone A., De Nigris B., Osanna M., Madariaga J.M., Maguregui M. (2021). When Red Turns Black: Influence of the 79 AD Volcanic Eruption and Burial Environment on the Blackening/Darkening of Pompeian Cinnabar. Anal. Chem..

[3] Bayarri V., Prada A., García F., Díaz-González L.M., De Las Heras C., Castillo E., Fatás P. (2023). Integration of Remote-Sensing Techniques for the Preventive Conservation of Paleolithic Cave Art in the Karst of the Altamira Cave. Remote Sens..

[4] Al-Omari A., Brunetaud X., Beck K., Al-Mukhtar M. (2014). Effect of thermal stress, condensation and freezing–thawing action on the degradation of stones on the Castle of Chambord, France. Environ. Earth Sci..

[5] Kamal H.M., Elkhial M.M., Tawfik T.S. (2018). The Role of Preventive Conservation in Designing 432 King Tutankhamun Galleries in the Grand Egyptian Museum. Stud. Conserv..

[6] Gil Cortés J. (2013). Diseño de un Sistema de Control de Temperatura y Humedad para la Conservación de puros 364 Habanos Mediante Controladores. http://hdl.handle.net/2099.1/19301.

[7] Una “Cápsula del Tiempo” en Madrid bajo la Estatua de Cervantes. https://www.larazon.es/madrid/capsula-tiempo-madrid-estatua-cervantes_2023031464101e6535808d0001cd427a.html.

[8] (1997). Climatic Conditions for Storage Environments of Graphic Documents and Features of the Housings.

[9] (1999). Properties of Historical and Artistic Interest—Environmental Conservation—Measurement and Analysis.

[10] (2010). Conservation of Cultural Property. Specifications for Temperature and Relative Humidity to Limit Climate-Induced Mechanical Damage in Organic Hygroscopic Materials.

[11] Innovative Packaging Solutions for Storage and Conservation of 20th Century Cultural Heritage of Artefacts Based on Cellulose Derivative. https://nemosineproject.eu/.

[12] Active & Intelligent Packaging Materials and Display Cases as a Tool for Preventive Conservation of Cultural Heritage. https://www.apacheproject.eu/.

[13] Innovative and Affordable Service for the Preventive Conservation Monitoring of Individual Cultural Artefacts during Display, Storage, Handling and Transport. https://www.collectioncare.eu/.

[14] Brunetaud X., de Luca L., Janvier-Badosa S., Beck K., Al-Mukhtar M. (2012). Application of digital techniques in monument preservation. Eur. J. Environ. Civ. Eng..

[15] Visco G., Plattner S.H., Fortini P., Di Giovanni S., Sammartino M.P. (2012). Microclimate monitoring in the Carcer Tullianum: Temporal and spatial correlation and gradients evidenced by multivariate analysis; first campaign. Chem. Cent. J..

[16] Onset U.S.A. Hobo Data Loggers. http://www.onsetcomp.com/.

[17] Merello P., García-Diego F.-J., Zarzo M. (2014). Diagnosis of abnormal patterns in multivariate microclimate monitoring: A case study of an open-air archaeological site in Pompeii (Italy). Sci. Total Environ..

[18] Temperature Logger iButton with 8KB Data-Log Memory. https://www.analog.com/en/products/ds1922t.html.

[19] Hygrochron Temperature/Humidity Logger iButton with 8KB Data-Log Memory. https://www.analog.com/en/products/ds1923.html.

[20] Merello P., Fernandez-Navajas A., Curiel-Esparza J., Zarzo M., Garcia-Diego F.-J. (2014). Characterisation of thermo-hygrometric conditions of an archaeological site affected by unlike boundary weather conditions. Build. Environ..

[21] Valero M.Á., Merello P., Fernández-Navajas Á., García-Diego F.-J. (2014). Statistical Tools Applied in the Characterisation and Evaluation of a Thermo-Hygrometric Corrective Action Carried out at the Noheda Archaeological Site (Noheda, Spain). Sensors.

[22] Lotrecchiano N., Sofia D., Giuliano A., Barletta D., Poletto M. (2019). Real-time On-road Monitoring Network of Air Quality. Chem. Eng. Trans..

[23] Verticchio E., Frasca F., Bertolin C., Siani A.M. (2021). Climate-induced risk for the preservation of paper collections: Comparative study among three historic libraries in Italy. Build Environ..

[24] García-Diego F.J., Zarzo M. (2010). Microclimate monitoring by multivariate statistical control: The renaissance frescoes of the Cathedral of Valencia (Spain). J. Cult. Herit..

[25] Fernández-Navajas Á., Merello P., Beltrán P., García-Diego F.-J. (2013). Software for Storage and Management of Microclimatic Data for Preventive Conservation of Cultural Heritage. Sensors.

[26] Diego F.-J.G., Esteban B., Merello P. (2015). Design of a Hybrid (Wired/Wireless) Acquisition Data System for Monitoring of Cultural Heritage Physical Parameters in Smart Cities. Sensors.

[27] Perles A., Pérez-Marín E., Mercado R., Segrelles J.D., Blanquer I., Zarzo M., García-Diego F.J. (2018). An energy-efficient internet of things (IoT) architecture for preventive conservation of cultural heritage. Future Gener. Comput. Syst..

[28] Botella J. (1995). El Sainet Fester.

[29] Fuentes Rodríguez C. (1986). Semiótica del sainete. Philol. Hispalensis.

[30] Vella R.C., Martinez F.J.R., Yousif C., Gatt D. (2020). A study of thermal comfort in naturally ventilated churches in a Mediterranean climate. Energy Build..

[31] Data Logger Wi-Fi Testo 160 THL. https://www.testo.com/es-ES/data-logger-wi-fi-testo-160-thl/p/0572-2024#tab-applications.

[32] EnviraIoT. https://enviraiot.es/monitorizacion-calidad-aire-museos/#.

[33] Conservación Preventiva en Museos y Edificios Históricos. https://sensonet.com/conservacion-preventiva-en-museos-y-edificios-historicos/.

